# Interventions to delay institutionalization of frail older persons: design of a longitudinal study in the home care setting

**DOI:** 10.1186/1471-2458-12-615

**Published:** 2012-08-06

**Authors:** Johanna De Almeida Mello, Therese Van Durme, Jean Macq, Anja Declercq

**Affiliations:** 1 PhD student Biomedical Sciences at LUCAS, KU Leuven, Leuven, Belgium; 2 Institute of Health and Society, Université catholique de Louvain, Louvain-la-Neuve, Belgium; 3 Institute of Health and Society, Université catholique de Louvain, Louvain-la-Neuve, Belgium; 4 Lucas and Center for Sociological Research, KU Leuven, Leuven, Belgium

**Keywords:** Innovative home care, Project evaluation, Frail older persons, Delay of institutionalization

## Abstract

**Background:**

Older people usually prefer staying at home rather than going into residential care. The Belgian National Institute for Health and Disability Insurance wishes to invest in home care by financing innovative projects that effectively help older people to stay at home longer. In this study protocol we describe the evaluation of 34 home care projects. These projects are clustered according to the type of their main intervention such as case management, night care, occupational therapy at home and psychological/psychosocial support. The main goal of this study is to identify which types of projects have the most effect in delaying institutionalization of frail older persons.

**Methods/design:**

This is a longitudinal intervention study based on a quasi-experimental design. Researchers use three comparison strategies to evaluate intervention - comparison among different types of projects, comparisons between older persons in the projects and older persons not benefiting from a project but who are still at home and between older persons in the projects and older persons who are already institutionalized. Projects are asked to include clients who are frail and at risk of institutionalization. In the study we use internationally validated instruments such as the interRAI Home Care instrument, the WHO-QOL-8 and the Zarit Burden Interview-12. These instruments are filled out at baseline, at exit from the project and 6 months after baseline. Additionally, caregivers have to do a follow-up every 6 months until exit from the project. Criteria to exit the cohort will be institutionalization longer than 3 months and death. The main analysis in the study consists of the calculation of incidence rates, cumulative incidence rates and hazard rates of definitive institutionalization through survival analyses for each type of project.

**Discussion:**

This research will provide knowledge on the functional status of frail older persons who are still living at home. This is important information to identify determinants of risk for institutionalization. The identification of effective home care projects in delaying institutionalization will be useful to inform and empower home care providers, policy and related decision makers to manage and improve home care services.

## Background

Older people usually prefer staying at home rather than going into residential care [1-3]. Several organizations and professionals provide care for older people at home. In 1994, the World Health Organization defined home care as “an array of health and social support services provided to clients in their own residence. The assumption is that these co-ordinated services may prevent, delay or be a substitute for temporary or long-term institutional care” [4]. A more recent definition of home care defines it as “a phenomenon in which care is provided by professionals to people in their own homes with the ultimate goal of not only contributing to their life quality and functional health status, but also to replace hospital care with care in the home for societal reasons”. In this definition, home care covers a wide range of activities, from preventive visits to end-of-life care [5]. According to the 2009 WHO-report, an appropriate balance between care settings for elderly care is necessary, including supported self-care and home-based service. The report emphasizes the need for interventions to help maintain older people at home and to prevent long-term institutional care [6].

In the literature, several studies about predictors for home care use can be found. The most predominant ones are age, living arrangement, number of informal caregivers, income, medical diagnosis and functional status [1,7]. Moreover, randomized controlled trials have shown that targeted home care can contribute significantly to reduce hospital admission, to delay institutionalization and to improve quality of life [8-10].

In Belgium, 125.000 older people received home care services in 2010 and by 2025 this number will have increased up to 149.000, according to population prognoses [11]. The Belgian National Institute for Health and Disability Insurance (NIHDI)^a^ wishes to invest in home care by financing innovative projects that effectively help older people to stay at home longer. In this study, the effects of these innovative projects are examined as to whether they do keep frail older people longer at home, whether they maintain or improve their quality of life and whether they preserve their autonomy [12-15].

Data from 34 innovative projects will be analyzed. Due to the fact that the projects vary in the scope of the intervention they provide, projects are clustered according to the type of their main intervention such as case management, night care, occupational therapy at home and psychological/psychosocial support. These projects might not be considered to be innovative in other countries. The term ‘innovative’ in the context of this research is defined as interventions which are not yet reimbursed by the Belgian NIHDI. These interventions can be seen as alternatives or add-ons to the standard home care services which are already funded (respite care, nursing care at home, physiotherapy, etc.).

The main goal of this study is to identify which types of projects have the most effect in delaying institutionalization of frail older persons.

## Methods

This is a longitudinal intervention study based on a quasi-experimental design [16]. Due to some constraints, a randomized controlled trial is impossible to realize. The constraints are mostly practical and ethical since all frail older people who wish to benefit from an innovative project cannot be denied access to it. In addition, researchers cannot have total control on the intervention since projects are delivering the services in the ‘real world’. Projects may organize their intervention according to specific types of services. Non randomization and variety of projects impose a thorough description and follow up of beneficiary population and intervention content. The Researchers use three comparison strategies to evaluate intervention – comparison of the arm benefiting from the intervention with older people benefitting from a different type of project, older people who are not benefiting from a project and are still living at home and older people who are already institutionalized.

### Typology of projects

Projects are clustered according to the type of intervention they perform (see Table 1). The typology definition is based on a previous study on the expressed intention and the perspective of actors involved in the project implementation [12].

**Table 1:** Typology of the projects according to main intervention

**Typology**	**Number of projects**
Case-management	8
Occupational therapy	7
Night care	10
Psychological or psychosocial support	9

### Sample size considerations

Beneficiaries will be grouped by type of projects as shown in Table 1 in order to get a reasonable sample size for each side of the comparison. Using an effect size of 0.50, a two-sided significance level of 0.05 and a power of 85%, 75 participants are needed in each group. We expect each project to include at least 45 participants per year because this is the minimum size of case load projects reported at the beginning of their activities. The clusters of projects will have then a larger sample size than indicated by the sample size calculation.

### Study population

The study will include older people receiving home care services who are at least 65 years old. Projects are asked to include only clients who are frail and at risk of institutionalization. Due to practical reasons, frailty is measured in the study with two different scales and caregivers are allowed to choose between them. One of these scales is the Edmonton Frail Scale [17,18] with a cutoff point of 6. Another scale is an adapted version of the Katz scale for Belgian home care and residential care [19]. Older people with a Katz score equal to A, B or C will be included [20]. Clients who have been diagnosed with dementia by a geriatrician, neurologist or psychiatrist may also be included in the study.

Each project has to collect data on at least 75% of their total client population in order to ensure representativeness. This means that caregivers have to complete questionnaires for at least 75% of clients benefiting from their projects.

### Ethical approval and informed consent

This observational study was approved by the Belgian Privacy Commission and by the Ethics committee of the Belgian Universities - Université Catholique de Louvain and Katholieke Universiteit Leuven with dossier number B40320108337. A formal procedure was implemented so that caregivers could fill out the questionnaires on a secured website.

For this study, older persons are asked to sign an informed consent agreement. In case they are not capable of signing this document, a family member or another legal representative will sign it on their behalf, as stipulated by Belgian law [21]. Clients do not undergo the intervention because of the research. The research however evaluates the effects of the intervention. Clients have the right not to participate in the research and they may withdraw their consent at any time. In that case, all data for this person is removed. Their consent or refusal to consent does not affect their participation in the intervention.

All data are anonymized and analyzed according to the rules of the Belgian Privacy Commission [22].

### Research questions

The aim of the study is to answer the following research questions:

1. Do characteristics of populations differ between projects?

2. Which types of projects are associated with less decrease of the functional and cognitive performance of frail older persons?

3. Which types of projects contribute to a better quality of life of frail older persons?

4. Which types of projects are associated with a decreased risk of institutionalization of frail older persons?

5. Is the interRAI Maple scale a good predictor of institutionalization in our study?

#### Selection of Variables

A previous systematic review identified the variables most frequently used in the evaluation of interventions aiming at maintaining frail older persons at home [14]. In our study, variables from the interRAI Home Care instrument which correspond to these identified variables will be used in the evaluation of interventions [23].

For data without any correspondence with the InterRAI instruments, other tools have been selected by a consensus amongst experts and according to their international validity. These complementary tools are the WHOQoL-8 and Zarit Burden scale (12-item) [24-26].

#### interRAI HC instrument

The interRAI HC instrument is an internationally validated instrument consisting of several domains such as cognitive functioning, ADL, social and psychological wellbeing, health status, environmental characteristics, etc. The use of a comprehensive geriatric assessment such as the interRAI HC is essential for this study in order to have an in-depth view of the client’s health and functional status [27-29]. The follow up of these characteristics will give insight in the evolution of the client’s situation and will enable researchers to show the effect of innovation projects. In the study we use the validated Belgian version of this instrument named BelRAI HC.

### WHO-QOL-8

The WHO defines Quality of Life as “individuals’ perception of their position in life in the context of the culture and value systems in which they live and in relation to their goals, expectations, standards and concerns” (24). The WHO-QOL-8 scale measures the perceived overall quality of life of the clients. It is derived from the WHO-QOL-Bref and contains each of the domains: physical and psychological health, social relations and environment [30,31]. It is currently used as an instrument in the WHO’s SAGE Survey [32]. This scale was designed for use where researchers need a short and concise instrument to measure quality of life. It is a self report instrument, so it has to be filled out by the clients themselves.

### Zarit Burden Interview 12 (ZBI-12)

In the international literature caregiver burden is perceived as a multidimensional response to stressors associated with care giving. These stressors may be physical, psychological, emotional, social and financial [33-35]. In this research we use a shorter version of the original 22-item scale Zarit Burden Interview. This scale contains 12 items with a score from 0 to 4. The total score can vary from 0 to 48 and the higher the total score, the higher the caregiver burden. The short form was chosen, in order not to put a heavier weight on the burden of the caregiver and to increase the response rate.

### Data collection

Professional caregivers are asked to fill out the interRAI Home Care instrument. Clients fill out the WHO-QOL-8 and the main informal caregivers complete the ZBI-12. These questionnaires are filled out at inclusion of the frail older person in the project (baseline), at exit from the project and 6 months after baseline. Additionally, if clients stay longer than 6 months in the project, caregivers have to do a follow-up every 6 months until the moment clients leave the project. Follow-up of clients happens from March 2011 until July 2013. A client follow-up period will vary between 6 to 36 months. Criteria to exit the cohort will be institutionalization longer than 3 months and death. Follow up will also end for clients who leave the projects.

Professional caregivers are instructed on how to fill out the questionnaires during a 2-day training and a follow up training of 1 day. They fill out the interRAI HC based on observation and on interview of the older person and of the main informal caregiver.

The interRAI-data will be linked with data from the national database IMA (Inter-mutuality Agency). This database contains data on national health service consumption reimbursed by NIHDI such as medication use, hospital days, medical, nursing and physical therapy use, etc. This data will be added to the analysis for the clients in the projects.

The “permanent sample” of people over 65 extracted from IMA database will also serve as a control group for our study population. By using this database we will be able to compare older persons who are benefiting from the innovative projects with older persons who are not benefiting from these projects (control group). In this control group we will have two major groups – clients who are still living at home and clients who are institutionalized. All clients who are in the projects and have not given their informed consent will be excluded from this database.

### Analyses

 – Research question 1 - Do characteristics of populations differ across projects?

In order to answer this first research question comparisons will be made between the populations in the different types of projects to explore whether they present particular characteristics. In these comparisons we will use the variables shown in Table 2 Frequencies will be calculated for categorical variables and means and medians with confidence intervals for continuous variables. Other possible comparison will be done by analysis of variance. All the statistical analysis in the study will be performed with the software Stata 11.1.

 – Research question 2 - Which types of projects are associated with less decline of the functional and cognitive performance of frail older persons?

 – Research question 3 - Which types of projects contribute to a better quality of life of frail older persons?

**Table 2:** Variables to characterize the population of the study

**Topic**	**Variable**	**Source**	**Type of variable**
General information	Civil status	InterRAI HC	Qualitative
	Age	InterRAI HC and IMA	Continuous
	Gender	InterRAI HC and IMA	Dichotomic
Quality of Life	WHOQOL items score	WHOQOL-8	Continuous
Socio-economic status	Financial difficulties	InterRAI HC	Dichotomic
Primary caregiver’s burden	Zarit Burden score	ZBI-12	Continuous
Functional status	Katz score	IMA	Qualitative
	interRAI ADL hierarchy scale	InterRAI HC	Ordinal (0 to 6)
	interRAI IADL scales	InterRAI HC	Ordinal (0 to 48)
	interRAI Communication scale	InterRAI HC	Ordinal (0 to 8)
	interRAI CPS2 scale	InterRAI HC	Ordinal (0 to 6)
	interRAI DRS scale	InterRAI HC	Ordinal (0-14)

Longitudinal analyses will be performed to answer the second and third research questions. Outcome variables will be the interRAI ADL hierarchy scale, the interRAI CPS2 scale and the WHOQOL scale. Mean will be compared between arms of the study and covariate analysis (ANCOVA) will be done to control results for potentially confounding variables.

 The ADL hierarchy scale is a measure of a person’s functional performance. It is based on the concept of early, middle and late loss of ADLs and consists of 4 items from the interRAI instruments: personal hygiene, toilet use, locomotion and eating. The scores vary from 0 to 6 and the higher the score, the greater the dependency [36,37].

 The cognitive performance scale (CPS2) is an internationally validated scale that describes a person’s cognitive status. The scores vary from 0 to 6 and the scale consists of 5 items from the interRAI HC instrument: skills for daily decision-making, making oneself understood, short-term memory recall, procedural memory and eating impairment. Higher scores indicate a greater degree of cognitive impairment. Several studies have shown a significant correlation between the CPS scale and the Mini-Mental State Examination (MMSE) [38-41].

 – Research question 4 - Which types of projects are associated with a decreased risk of institutionalization of frail older persons?

In order to answer the fourth research question, the definitive institutionalization (more than 90 consecutive days in residential care) will be used as event. The analysis consists of the calculation of incidence rates, cumulative incidence and hazard rates of definitive institutionalization through survival analyses for each type of project. Survival analysis measures the time to a certain event or change of state, in this case, institutionalization. The multivariate Cox model which will be calculated from this analysis will allow us to isolate the effects of the intervention from the effects of confounding variables. These variables are extraneous variables which correlate (positively or negatively) with both the dependent variable and the independent variable. In this analysis we will use the variables of Table 2 as confounding variables. The use of a Cox model may improve the estimate of the intervention effect by narrowing the confidence interval. Another important advantage of this analysis is that it takes into account the variability of covariates across time which makes it very appropriate for longitudinal studies [42-44].

 – Research question 5 - Is the interRAI Maple scale a good predictor of institutionalization in our study?

The fifth research question will be approached by using a multiple logistic regression model with the Methods for Assigning Priority Levels scale (Maple) as one of the independent variables. This interRAI scale is known to be a predictor for institutionalization and can be used as an indicator for allocation of home care resources and prioritization of clients needing community or residential care. MAPLe is an internationally validated predictor of nursing home placements, caregiver distress and ratings that the client would be better off elsewhere [45].

## Discussion

This paper describes the study protocol of a longitudinal quasi-experimental research to evaluate the effect of innovative projects in delaying institutionalization of frail older persons.

A major strength of the study is the follow up period of 3 years, which allows researchers to obtain a large sample size and several measurement points for a number of clients. Most clients will have at least 3 measurements. Another major strength is the availability of the national database IMA to enable researchers to have a large control group and to have data on health services and medication consumption of the study population. In addition, the use of international validated instruments such as the ZB-12 item, the WHOQOL 8 and the interRAI HC will also make further cross-national comparisons possible.

Some limitations of the study are due to the lack of randomization in the allocation between projects and due to the fact that not all variables collected in the population of the projects will be found in the control group of older persons not benefiting from the projects.

The research will provide knowledge on the functional situation of frail older persons who are still living at home and will allow researchers to make comparisons with older persons who are already institutionalized. This is important information to identify determinants of risk for institutionalization. The identification of effective projects in delaying institutionalization will be useful to inform and empower home care providers, policy and related decision makers to manage and improve home care services.

## Endnote

^a^The NIHDI is a Belgian federal institution which manages and supervises the correct application of the health insurance in Belgium.

## Abbreviations

WHO: World Health Organization; NIHDI: National Institute for Health and Disability Insurance; WHO-QOL-8: World Health Organization Quality of Life instrument 8; ZBI 12: Zarit Burden Interview - 12 items; interRAI HC: interRAI Home Care instrument; IMA: Inter-Mutuality Agency; ADL: Activities of Daily Living; CPS2: InterRAI Cognitive Performance scale 2; IADL: Instrumental Activities of Daily Living; MMSE: Mini-Mental State Examination; sDRS: interRAI Depression Rating Scale.

## Competing interests

The authors declare that they have no competing interests.

## Authors’ contributions

dAMJ, VDT, MJ and DA are involved in the study design and critically reviewed and approved the final manuscript. dAMJ drafted the manuscript. All authors read and approved the final manuscript.

## Pre-publication history

The pre-publication history for this paper can be accessed here:

http://www.biomedcentral.com/1471-2458/12/615/prepub
